# Trained immunity as a potential target for therapeutic immunomodulation in Duchenne muscular dystrophy

**DOI:** 10.3389/fimmu.2023.1183066

**Published:** 2023-06-15

**Authors:** Basil J. Petrof, Tom Podolsky, Salyan Bhattarai, Jiahui Tan, Jun Ding

**Affiliations:** ^1^ Meakins-Christie Laboratories, Translational Research in Respiratory Diseases Program, Research Institute of the McGill University Health Centre, Montreal, QC, Canada; ^2^ Department of Medicine, McGill University Health Centre, Montreal, QC, Canada; ^3^ Department of Biostatistics and Systems Biology, School of Public Health, Sun Yat-sen University, Shenzhen, China

**Keywords:** innate immunity, macrophages, muscular dystrophies, mdx mouse, sterile inflammation, chronic inflammatory diseases, immune memory

## Abstract

Dysregulated inflammation involving innate immune cells, particularly of the monocyte/macrophage lineage, is a key contributor to the pathogenesis of Duchenne muscular dystrophy (DMD). Trained immunity is an evolutionarily ancient protective mechanism against infection, in which epigenetic and metabolic alterations confer non-specific hyperresponsiveness of innate immune cells to various stimuli. Recent work in an animal model of DMD (mdx mice) has shown that macrophages exhibit cardinal features of trained immunity, including the presence of innate immune system “memory”. The latter is reflected by epigenetic changes and durable transmissibility of the trained phenotype to healthy non-dystrophic mice by bone marrow transplantation. Mechanistically, it is suggested that a Toll-like receptor (TLR) 4-regulated, memory-like capacity of innate immunity is induced at the level of the bone marrow by factors released from the damaged muscles, leading to exaggerated upregulation of both pro- and anti-inflammatory genes. Here we propose a conceptual framework for the involvement of trained immunity in DMD pathogenesis and its potential to serve as a new therapeutic target.

## Introduction

The genetic basis for Duchenne Muscular Dystrophy (DMD) has been known for over 30 years (1), but despite tremendous progress in the field its pathophysiology remains incompletely understood. The disease is caused by mutations in the *DMD* gene on the X chromosome encoding dystrophin, a large (427 kD) membrane-associated cytoskeletal protein (2). In the absence of dystrophin, muscle fibers suffer repetitive damage which eventually leads to their replacement by fibrosis and adipose tissue. Among several established and putative functions for dystrophin (3), a primary role is to protect the muscle cell surface membrane (sarcolemma) from contraction-induced mechanical injury (4, 5). Exaggerated pro-inflammatory signaling in skeletal muscle is observed shortly after birth and is a major contributor to disease progression (6–11). Corticosteroids, although only transiently effective and associated with significant side effects, are currently the main pharmacologic therapy in DMD patients (12).

In animal models of DMD such as the mdx mouse, specific interference with the function of several inflammatory cell subtypes and pro-inflammatory mediators has shown beneficial effects, particularly during early stages of disease (8–11). However, there is also evidence that co-existent and overly vigorous anti-inflammatory signaling promotes the fibrosis and muscle regeneration failure which are characteristic of the later stages of pathology (6, 13–16). This evolving landscape, with combined induction of pro-inflammatory as well as anti-inflammatory signaling, creates a moving target that complicates decisions about the optimal timing for instituting (or withdrawing) different potential immunomodulatory therapies. The ideal immunotherapy for DMD would have a dual ability to both dampen injurious inflammation and prevent a disproportionate “overshoot” of anti-inflammatory responses. In this review we discuss the possibility that one potential avenue for achieving this goal is through therapeutic targeting of trained immunity, a form of innate immune memory that has recently been implicated in the pathogenesis of several chronic inflammatory diseases, including DMD.

## Role of innate immunity in DMD

There have been several recent excellent reviews of the role of immune cells in skeletal muscle injury and muscular dystrophy (8–11, 17). In human DMD patients as well as mdx mice (the most commonly used pre-clinical model of DMD), myeloid cells of the monocyte/macrophage (MP) lineage are the most abundant inflammatory cell type within the dystrophic skeletal muscles (18, 19). Although other myeloid cell types such as mast cells (20) and eosinophils (21, 22) may also play roles in DMD pathogenesis, they are present in much lower numbers. We and others have shown that prevention of monocyte/MP recruitment to mdx muscles significantly improves muscle fiber pathology and strength in early disease (18, 23). Much research has focused on the fact that MPs demonstrate a high degree of plasticity and can exhibit pro-inflammatory (classically activated M1, M1-biased, or M1-like) or anti-inflammatory (alternatively activated M2, M2-biased, or M2-like) properties in skeletal muscle (8–11). In general, prototypical M1 MPs produce elevated levels of pro-inflammatory cytokines as well as inducible nitric oxide synthase (NOS2) and reactive oxygen/nitrogen species. Prototypical M2 MPs express high levels of anti-inflammatory cytokines along with increased quantities of different scavenger receptors. Pro-inflammatory MPs stimulate the proliferation of myogenic precursor (satellite) cells and inhibit their differentiation, whereas anti-inflammatory MPs have the reverse effect (24, 25). Although it is well recognized that the pro-inflammatory/M1 versus anti-inflammatory/M2 framework vastly oversimplifies the degree and spectrum of MP heterogeneity (26–28), this nomenclature is nonetheless useful for conceptual purposes and will be referred to throughout this review.

In vitro, a large number of soluble factors can preferentially drive MPs toward either M1 or M2 phenotypic features (29–32). In vivo, M1-biased MPs are believed to exacerbate muscle damage in DMD through release of nitric oxide and other pro-inflammatory mediators (18, 33, 34), whereas M2-biased MPs (with an increased ratio of TGFβ to TNF production) promote the survival of fibroadipogenic progenitor cells and favour the development of fibrosis (13, 15). However, multiple mediators having myriad and sometimes opposing effects, and arising from different cell types, are often simultaneously present within the same tissue microenvironment (8, 16). The repetitive episodes of muscle injury in DMD occur in a manner that is both temporally and spatially asynchronous (35). Under these conditions, M1- and M2-biased MPs can co-exist alongside one another and send conflicting signals to the repairing muscle (8, 16). In addition, flow cytometry has revealed substantial numbers of MPs in dystrophic muscles which do not fit well into the canonical M1-M2 polarization paradigm (15, 23, 36–38). For example, MPs expressing Ly6C (generally considered a marker of recently recruited pro-inflammatory monocytes) also express elevated levels of TGF-β in mdx muscles (38). Recent single cell RNA sequencing studies have further demonstrated the complexity and heterogeneity of MP populations within dystrophic skeletal muscles of both mice and humans (39, 40). This includes the presence of MPs with a mixed M1/M2 gene expression pattern within the same cell population, which are rarely observed in healthy muscle and may represent maladaptive immune cell dysregulation. As discussed below, trained immunity could play an important role in promoting the generation of such dysregulated MP populations in DMD, leading to functional alterations which favour injury and/or impede effective muscle regeneration.

## What is trained immunity?

The immune system is traditionally divided into two main arms, innate and adaptive immunity. Innate immunity is an evolutionarily ancient phenomenon, whereas adaptive immunity is more recent and unique to vertebrate species. Innate immunity is largely mediated by myeloid cells such as monocytes and MPs, neutrophils, eosinophils, mast cells, and basophils. These cells respond very rapidly to threats sensed via pattern recognition receptors (eg., Toll-like receptors, TLRs) and other mechanisms, and represent the first line of defense while awaiting arrival of the more slowly developing adaptive immune response. The latter employs immunoglobulin gene recombination and clonal expansion in B and T lymphocytes to confer long-lasting immunological memory of specific antigens. In contrast, the innate immune system is classically considered to lack both long-term memory and antigen-specificity. However, in recent years there has been increasing recognition that invertebrates devoid of an adaptive immune system, as well as immunocompromised mammals with severe defects in adaptive immunity, are nonetheless capable of mounting a form of innate immune cell memory (41–43), now commonly referred to as trained immunity (44).

Many experimental studies have documented the existence of such innate immune memory and its ability to protect against future infections (44). Importantly, this protection is afforded not only against the microbe that initially triggered this response, but also for later infections by completely unrelated organisms. For example, induction of trained immunity by exposure of mice to the fungal pathogen *Candida albicans* provides protection against subsequent bacterial infection by *Staphylococcus aureus* (42). Along the same lines, administration of the bacillus Calmette-Guerin (BCG) vaccine against tuberculosis to severe combined immunodeficiency (SCID) mice, which lack functional B and T lymphocytes, induces trained immunity and protects against later infection by *Candida albicans* (45). The fungal wall component beta-glucan and BCG-related ligands for cellular pattern recognition receptors are two examples of specific microbial products capable of inducing trained immunity (45, 46). In addition to such exogenous microbial components (pathogen-associated molecular patterns, PAMPs), several endogenous ligands (damage-associated molecular patterns, DAMPs) that are increased in chronic non-communicable diseases have also been reported to induce trained immunity. Examples of such DAMPs include oxidized low-density lipoproteins, lipoprotein (a), aldosterone, catecholamines, and uric acid (47–51). However, in contrast to the generally beneficial effects of trained immunity in the context of infections, it has been proposed that trained immunity induced by DAMPs could be an important contributor to dysregulated inflammation driving progression of chronic non-infectious diseases such as atherosclerosis (48, 52–54), Alzheimer’s disease (55), chronic allergy (56–59), organ transplant dysfunction (60), diabetes (61–64), and retinal degeneration (65).

At the cellular level, the trained immunity phenotype displays a number of characteristic features (44, 66). Fundamentally, exposure to a primary stimulus reprograms the cells to develop an exaggerated transcriptional response of innate immunity genes upon subsequent re-challenge with the same agent. However, in contrast to adaptive immunity which is antigen-specific, the above transcriptional hyperresponsiveness upon re-challenge is not specific to the original inciting agent and can also be triggered by exposure to antigenically unrelated stimuli. Epigenetic alterations that favour a more open chromatin state and permit greater accessibility of transcription factors to the DNA regulatory elements of innate immune system genes account for the enhanced transcriptional responses which occur upon re-challenge, as well as its long-lasting nature. Finally, the trained cells typically demonstrate metabolic changes consisting of upregulated glycolytic (and in some cases oxidative phosphorylation) pathways, which provide the energy and substrates required for the above cellular responses.

Evidence of trained immunity has been reported in diverse immune cell types including monocytes, MPs, neutrophils, dendritic cells, innate lymphoid cells, and natural killer cells, as well as in several non-immune cell types (eg. epithelial, endothelial, fibroblasts, smooth muscle) (44). However, the majority of studies describing the features and mechanisms underlying trained immunity have been performed in monocyte/MP lineage cells. Given that innate immune cells in the blood typically turn over after only a few days, the observation that monocytes exhibiting the characteristics of trained immunity can persist for months after exposure to an inducing stimulus [eg. for 3 months after BCG vaccination in humans (45, 67)], led to the hypothesis that epigenetic reprogramming must take place at the level of myeloid progenitor cells in the bone marrow compartment. This mechanism, referred to as central induction of trained immunity, has been confirmed by elegant studies performed in both mice and humans (67–70). In addition, there is accumulating evidence that trained immunity can also be induced in long-lived tissue resident immune cells which are not derived from the bone marrow, such as brain microglia (55) and alveolar MPs (71).

## Evidence for trained immunity in DMD

To explore whether trained immunity might play a role in DMD, we recently examined the functional and epigenetic status of MPs derived from myeloid progenitors in the bone marrow of mdx mice (72). Bone marrow-derived macrophages (BMDM) from mdx mice were exposed in vitro to a variety of distinct provocative stimuli. The mdx BMDM demonstrated exaggerated upregulation of multiple innate immune response genes in comparison to equivalently treated BMDM from non-dystrophic wild-type mice. This hyperresponsiveness of mdx BMDM was non-specific as it was found after exposure to different cytokines (IFNγ, IL-4) as well as structurally unrelated DAMPs (fibrinogen, beta-glucan). As discussed earlier, this broadly increased responsiveness to multiple forms of stimulation is a cardinal feature of trained immunity. In addition, mdx BMDM exhibited a basal upregulation of multiple innate immune genes as well as major metabolic alterations consisting of lower oxygen consumption and increased lactate production by mdx BMDM during the early necrotic phase of the disease. Interestingly, mdx BMDM at the more advanced fibrotic stage of disease showed a reversal of this pattern with a more oxidative profile compared to age-matched wild-type mice. To the extent that M2-biased MPs are more reliant on oxidative phosphorylation (73), these findings could help to explain the previous observation that intramuscular MPs evolve from predominately M1-biased toward a more M2-biased phenotype over the course of disease progression (13, 14, 34).

Nucleosomes comprise the basic subunit of chromatin, and are composed of the histone proteins H2A, H2B, H3, and H4. The amino-terminal tails of these histones are subject to a wide variety of post-translational modifications (“histone marks”), such as acetylation (ac) and methylation (me), which alter chromatin functionality and transcriptional activity. Studies in trained immune cells have reported a range of histone modifications such as increased levels of histone H3 lysine-27 acetylation (H3K27ac) and histone H3 lysine-4 monomethylation (H3K4me1) or trimethylation (H3K4me3), which are all classically associated with enhanced chromatin accessibility and transcriptional activation (41, 42, 48, 74). In addition, the demethylase Jmjd3 was reported to reduce levels of the repressive histone mark H3K27me3 at the IL-12 promoter in BMDM from diabetic mice exhibiting features of trained immunity (63). KDM4 histone demethylases, which similarly remove methylation marks linked to transcriptional repression such as histone H3 lysine-9 trimethylation (H3K9me3), have also been identified as potential regulators of trained immunity in human monocytes (75).

To explore whether such epigenetic alterations occur in mdx mice, chromatin immunoprecipitation (ChIP) analyses of histone modifications were performed in mdx BMDM (72). Genome-wide ChIP-seq revealed that the repressive histone mark H3K27me3 was reduced in mdx BMDM, which is in line with the more open chromatin state and enhanced gene transcription characteristic of trained immunity. This decrease of H3K27me3 in mdx BMDM involved key pro-inflammatory, anti-inflammatory, and pro-fibrotic genes associated with dystrophic muscle pathology. ChIP-PCR confirmed these findings and additionally indicated increased levels of the activating histone mark H3K4me3 on both M1- and M2-biased gene promoters. Interestingly, the mdx BMDM also exhibited a decrease in the activating histone mark H3K27ac, a change which would in contrast be expected to reduce the level of gene transcription. Pathway analysis suggested that decreases in the H3K27ac mark in mdx BMDM preferentially involved biological processes associated with protein metabolism, the cell cycle, and the regulation of gene expression. However, for the pro-inflammatory, anti-inflammatory, and pro-fibrotic genes potentially involved in DMD pathogenesis, the ratio of acetylation to trimethylation of H3K27 was increased which is in keeping with a more open chromatin state. In addition to the demonstration of histone mark alterations, the presence of epigenetic imprinting in bone marrow monocyte/MP myeloid precursors of mdx mice is strongly supported by chimeric experiments in which the hematopoietic stem cell compartment of irradiated wild-type mice was reconstituted with transplanted mdx mouse bone marrow (72). Under these conditions, the trained immunophenotype of transplanted mdx donor origin BMDM remained intact despite being placed in normal recipient mice without muscular dystrophy. This durability of the phenotype in mdx origin BMDM was maintained for at least 3 months after transplantation into the non-dystrophic host environment, which is in keeping with long-lasting epigenetic reprogramming of the cells.

**Figure 1:**
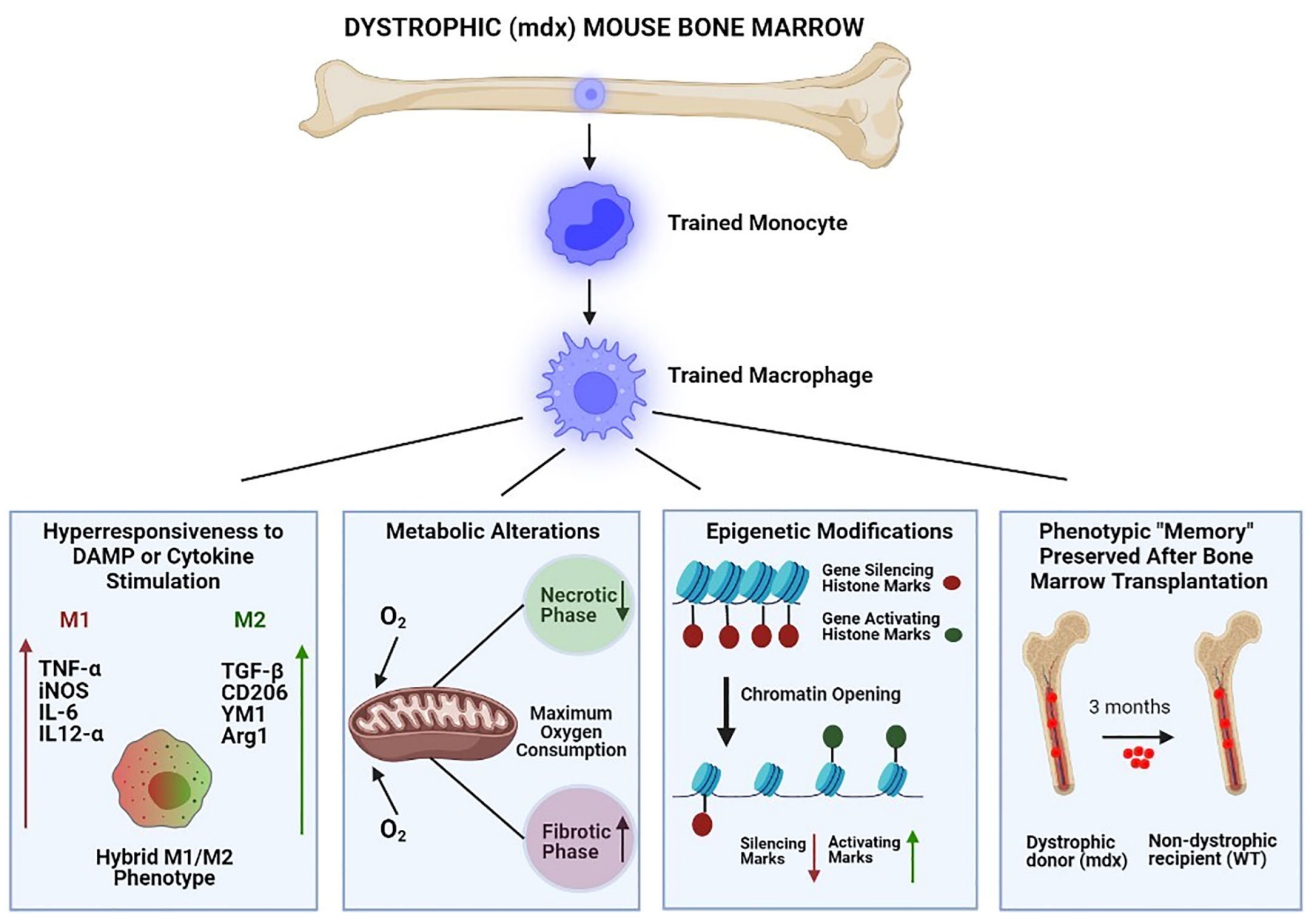
Bone marrow-derived macrophages of dystrophic (mdx) mice demonstrate keystone features of trained immunity. From left to right: 1) Hyperresponsiveness to stimulation by damage-associated molecular pattern (DAMP) molecules and cytokines, resulting in exaggerated innate immune gene expression (both pro-inflammatory/M1 and anti-inflammatory/M2); 2) Metabolic alterations (e.g. maximal oxygen consumption) which vary according to disease stage (necrotic versus fibrotic phase); 3) Epigenetic modifications which increase the chromatin accessibility of innate immune system genes by reducing gene-silencing and increasing gene-activating histone marks; and 4) Innate immune system “memory” characterized by durable (3 months) maintenance of the hyperresponsive phenotype after mdx bone marrow engraftment into non-dystrophic wild-type (WT) mice.

In summary, monocyte/MP lineage cells of mdx mice demonstrate hallmark features of trained immunity including: 1) increased DAMP and cytokine-stimulated innate immunity gene expression, 2) changes in cellular metabolism, 3) epigenetic remodeling, and 4) innate immune “memory” characterized by maintenance of a transcriptionally hyperresponsive phenotype even after long-term removal from the muscular dystrophy environment through bone marrow engraftment into non-dystrophic mice (**Figure 1**).

## Potential stimuli for trained immunity in DMD: the role of TLR4

The ability of an intrinsic muscle disease such as DMD to induce trained immunity at the level of the bone marrow suggests that one or more factors released from the injured muscles, such as DAMPs, could act as primary inciting stimuli *in vivo*. Previous work has shown that low-dose exposure to the TLR4 ligand lipopolysaccharide (LPS) can induce trained immunity in human monocytes (76). For example, systemic LPS administration induced trained immunity in brain microglial cells that persisted for at least 6 months in mice (55). In another study, transient LPS exposure in mice led to persistent alterations of myeloid enhancer accessibility within hematopoietic stem cells, accompanied by improved innate immunity against infection (77). Along the same lines, recent work has implicated the TLR adaptor protein, MyD88, in the generation of trained immunity in murine MPs following exposure to different DAMPs (78).

We previously reported that global TLR4 deficiency in mdx mice reduces the number of pro-inflammatory MPs as well as other pathological features within dystrophic muscles (37). This is consistent with a study demonstrating that genetic abrogation of MyD88 also improves skeletal and cardiac muscle pathology in mdx mice (79). Among the DAMPs that are chronically increased in the muscles and/or serum of mdx mice and DMD patients (80, 81), fibrinogen has been directly implicated in disease progression and serves an endogenous TLR4 ligand (13, 82). Several other DAMPs can also act as endogenous ligands for TLR4 (83). In addition to the direct cellular effects of TLR4 engagement by various DAMPs, other factors released from diseased dystrophic muscles such as cytokines (55) or exosomes (84) could also play significant roles in the generation of trained immunity in a manner that is either dependent or independent of TLR signaling.

To determine whether endogenous factors derived from damaged muscle can induce the trained immunity phenotype, BMDM from healthy wild-type mice were exposed to crushed skeletal muscle extract as a primary training stimulus (72). These cells demonstrated exaggerated upregulation of innate immune system genes when secondarily re-challenged 5 days later with different structurally diverse DAMPs. Importantly, the potentiated responses to secondary stimulation observed in wild-type BMDM exposed to crushed muscle extract were lost in the absence of TLR4. To more specifically explore the role of TLR4 in the development of trained immunity in DMD, key features of the trained immune response were examined in mdx mice with genetic deficiency of TLR4 (mdxTLR4^-/-^) (72). In mdxTLR4^-/-^ BMDM, both transcriptional hyperresponsiveness to heterologous forms of stimulation and the altered metabolic phenotype observed in mdx BMDM were eliminated.

**Figure 2:**
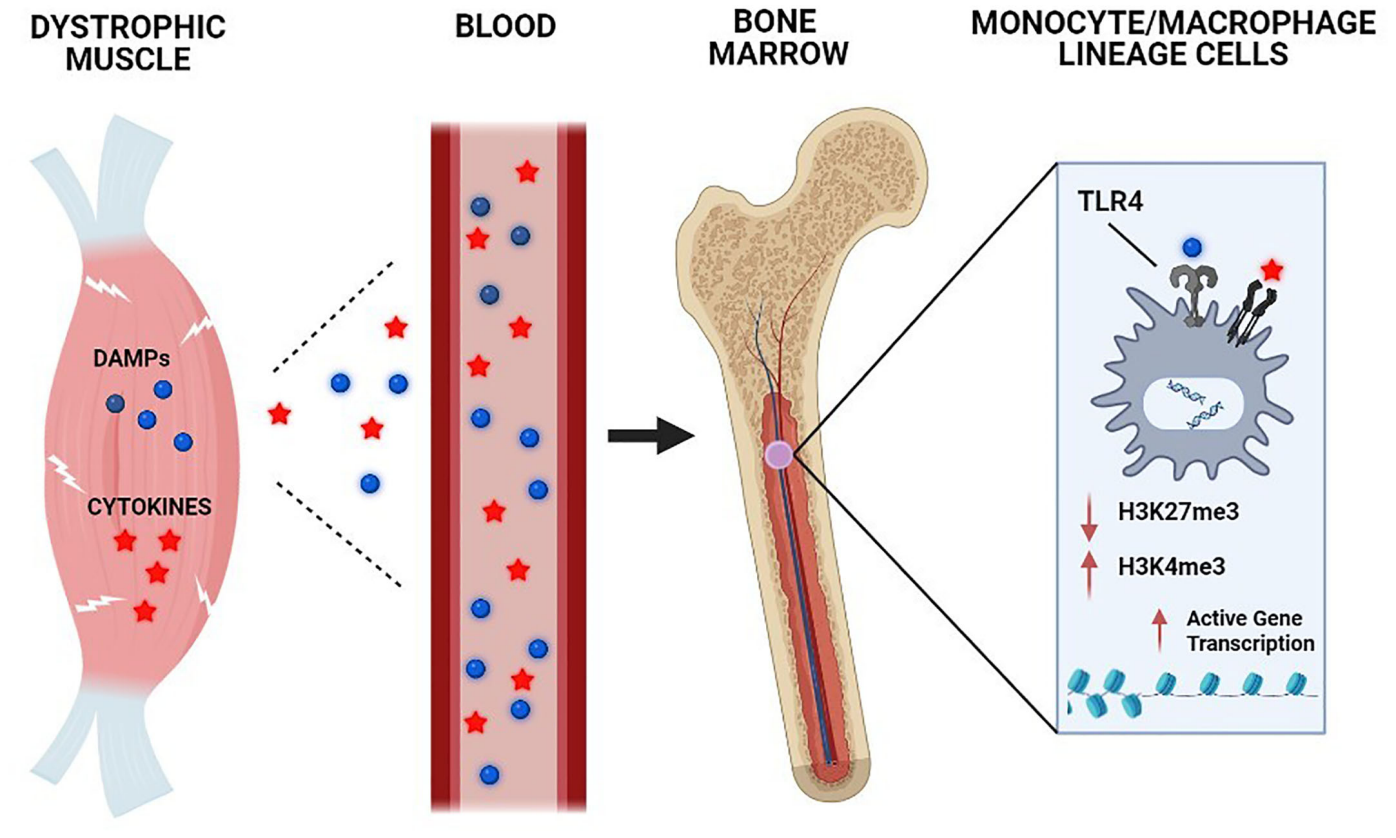
Chronic skeletal muscle damage in muscular dystrophy causes the release of systemic mediators capable of inducing trained immunity. The underlying genetic defect in DMD leads to chronic skeletal muscle injury, which results in the systemic release of damage-associated molecular pattern (DAMP) molecules and cytokines. These circulating factors in the bloodstream are transported to the bone marrow compartment, where they act on monocyte/macrophage lineage cells through Toll-like receptor (TLR) 4 and other receptors to induce epigenetic remodeling and other key aspects of trained immunity.

The impact of TLR4 deficiency on features of trained immunity in mdx mice was also reflected at the epigenetic level. ChIP-seq revealed that the predominant H3K27me3 pattern found in mdx BMDM (reduced compared to wild-type) was prevented by the absence of TLR4, leading to restoration of H3K27me3 levels in the mdxTLR4^-/-^ group. On the other hand, the general reduction of H3K27ac signal intensity observed in mdx BMDM was further amplified in the mdxTLR4^-/-^ BMDM. Hence, the histone modifications demonstrated in mdx mice lacking TLR4 (increased H3K27me3 and decreased H3K27ac) would both be expected to decrease chromatin accessibility and transcriptional activity. Overall, the results of these studies collectively support the concept that TLR4 activation and signaling in monocyte/MP lineage cells, most likely in response to DAMP release from injured skeletal muscles, plays a role in the development of trained immunity in muscular dystrophy (**Figure 2**).

## Implications of trained immunity for DMD pathogenesis and treatment

**Figure 3:**
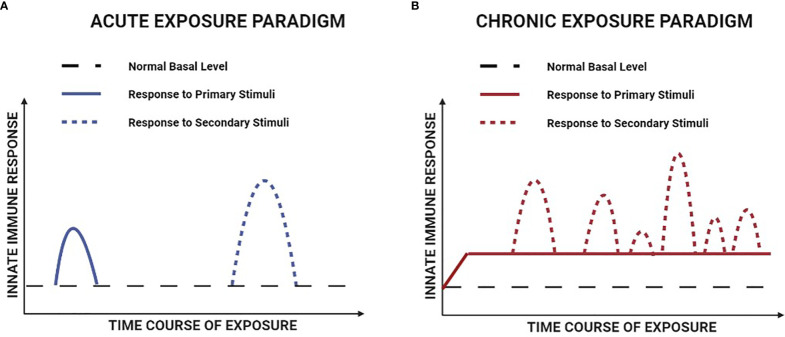
Hypothetical comparison of the responses to primary and secondary stimuli in acute versus chronic exposure paradigms of innate immune memory. **(A)** For innate immune memory induced by an acute exposure paradigm (e.g. acute infection or vaccination), the primary and secondary stimuli are both transiently present and there is a return of immune gene transcription to normal basal levels between the two stimulation events. **(B)** For innate immune memory induced by the chronic disease paradigm of DMD, immune gene transcription is persistently elevated over time, due to the combination of continuous background exposure to primary stimuli and intermittent episodes of superimposed secondary stimulation.

In the case of trained immunity induced by transient exposure to an acute infection or vaccination, the initial increase in innate immune gene transcription typically subsides and returns to normal basal levels once the primary inciting stimulus has been removed (85). However, long-lasting epigenetic alterations ensure the ability to produce a more robust response to secondary stimuli at a future date. It is important to recognize that this scenario differs fundamentally from most chronic non-infectious diseases, where the primary inciting stimulus is derived from the underlying disease process and thus present on a continuous basis. This is the case in DMD, where elevated levels of innate immune gene expression induced by chronic background exposure to muscle damage factors are likely to be potentiated by intermittent periods of more intense muscle necrosis (**Figure 3**). To distinguish between acute and chronic exposure scenarios, it has been suggested that the latter situation be referred to as adaptation (66) or priming (85). Irrespective of the nomenclature employed, the fact that the hyperresponsive phenotype of mdx mice is associated with epigenetic changes and maintained for months after heterologous bone marrow transplantation is consistent with innate immune system memory. Furthermore, in an analogous fashion to autoimmune disorders in which adaptive immune system memory is continually stimulated and thereby promotes disease progression, chronic stimulation of innate immune system memory could similarly contribute to the maintenance of pathological inflammation in DMD.

The studies of mdx mice indicate that even before their recruitment to dystrophic muscle, future monocyte/MPs present within the bone marrow have already undergone extensive epigenetic and functional remodeling. In addition, cultured mdx BMDM show simultaneous increases in the expression of both M1 and M2 marker genes rather than simple skewing toward M1- or M2-biased profiles (72), a finding which has been reported in other examples of trained immunity (42, 74). This pattern is consistent with the characteristics of intramuscular MPs found within dystrophic muscles *in vivo*, where one also finds an increased proportion of MPs exhibiting combined upregulation of both M1 and M2 genes (23, 36–38). We speculate that the hybrid M1/M2 phenotype of MPs from the mdx bone marrow results from the observed epigenetic reprogramming, which places the cells in a state that is more conducive for rapidly assuming different possible phenotypes (M1-biased, M2-biased, or some combination thereof) once they are recruited to the muscle tissue. In principle this would have the advantage of allowing for more efficient adaptation to the microenvironmental conditions encountered within the muscle. In the setting of chronic disease with ongoing asynchronous muscle injury, however, this property may be maladaptive and lead to chaotic or conflicting molecular signaling with adverse consequences for muscle repair (16, 35). Such aberrant molecular signals are prime candidates for triggering an exaggerated “overshoot” of either M1 or M2 gene expression by trained MPs within the dystrophic muscle microenvironment. Indeed, an inappropriate balance between pro-inflammatory (TNF) and anti-inflammatory (TGF-β) cytokine expression within hybrid MPs has been shown to be an important driver of dystrophic pathology (15).

**Figure 4:**
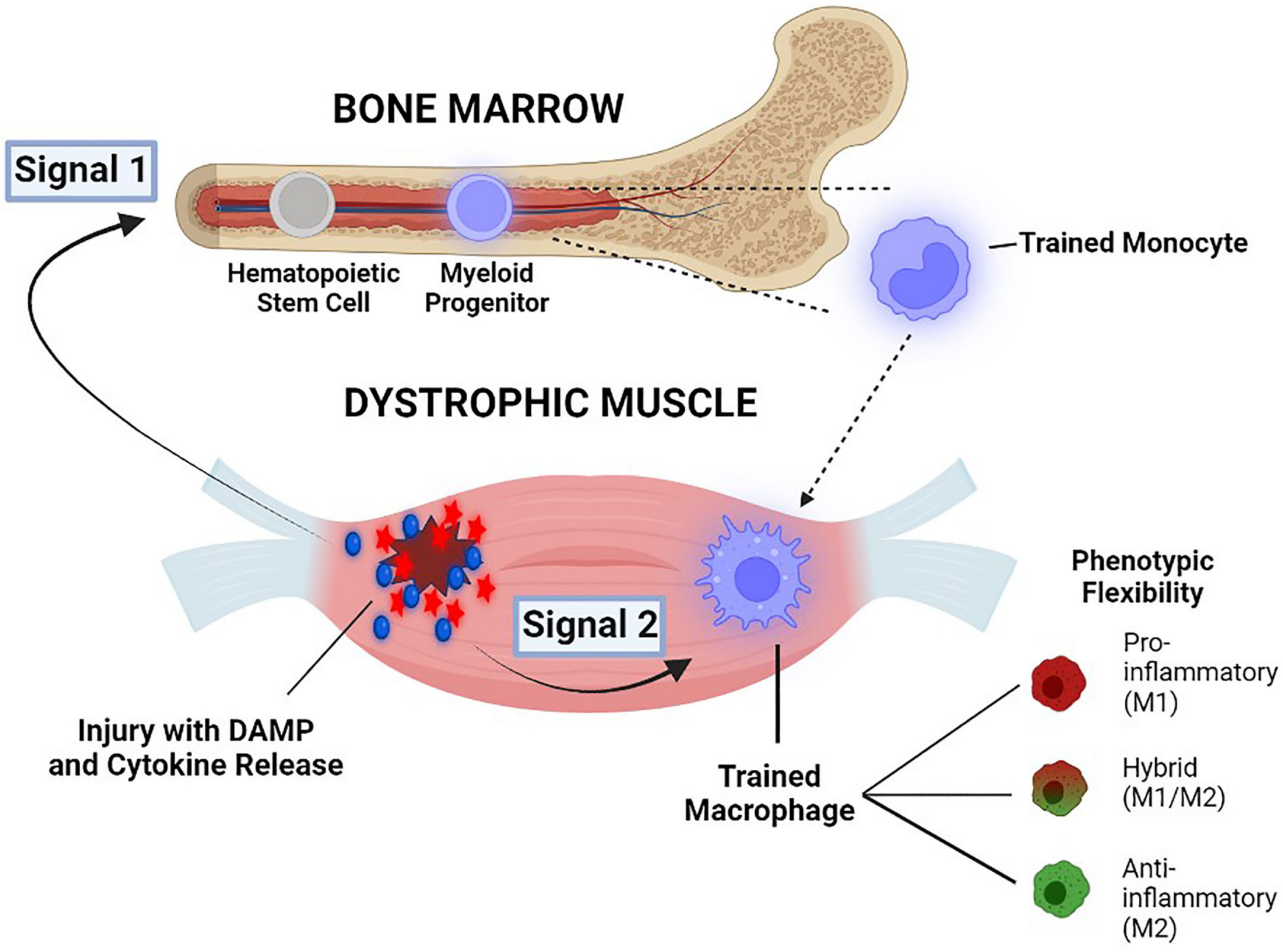
Illustration of a two-signal, two-compartment model to conceptualize the possible role of trained immunity in DMD pathogenesis. Damage-associated molecular pattern (DAMP) molecules and cytokines which are released into the blood circulation from dystrophic muscles, serve as primary stimuli for the induction of trained immunity in the bone marrow compartment (Signal 1). Trained monocytes are then recruited to the pathological microenvironment of the dystrophic muscle compartment, where they encounter either the same or different muscle-derived factors that serve as potentiating secondary stimuli (Signal 2). The more open chromatin state for both pro-inflammatory/M1 and anti-inflammatory/M2 genes allows for phenotypic flexibility of MPs (M1-biased, M2-biased, or non-classifiable hybrid phenotypes) See text for further explanation.

As a hypothetical framework for future investigation, we propose a two-signal, two-compartment model to conceptualize the possible sequence of pathological events involving trained immunity in DMD (**Figure 4**). We posit that ongoing muscle necrosis present from the earliest stages of DMD acts as the source of low levels of DAMPs, cytokines and other molecules which serve as primary stimuli for the induction of trained immunity within monocytes in the bone marrow compartment (termed Signal 1). This triggers the epigenetic and metabolic changes in these cells that create a state of heightened innate immune system “readiness”, with an inherently greater ability to upregulate both M1-biased (pro-inflammatory) and M2-biased (anti-inflammatory) genes. Trained monocytes harbouring this broad potential for enhanced transcription of innate immune system genes are subsequently recruited to the dystrophic muscles where they undergo differentiation into MPs. Within this pathological intramuscular environment, the monocyte-derived MPs are exposed to either the same or different muscle-derived DAMPs, cytokines, and other factors, which can now serve as potentiating secondary stimuli within the skeletal muscle compartment (termed Signal 2). Hence we hypothesize that Signal 1-induced reprogramming of monocytes centrally in the bone marrow induces the increased potential of these cells to rapidly adopt different possible phenotypes, whereas Signal 2 factors encountered peripherally in the dystrophic muscle milieu are largely responsible for directing this enhanced potential toward the final phenotypic outcome. The nature and magnitude of contributions from both Signal 1 and Signal 2 would be expected to vary at different stages of disease. Importantly, according to this model the intramuscular MP phenotype driven by trained immunity is flexible and could be M1-biased, M2-biased or a non-classifiable hybrid phenotype.

In conclusion, overly exuberant and dysregulated MP responses associated with trained immunity may contribute to the counter-productive inflammatory milieu that impedes successful muscle regeneration in DMD. Accordingly, future investigations should explore whether prevention or reversal of trained immunity is capable of favorably modifying the course of disease in DMD. Because DAMP-mediated TLR4 signaling appears to play an important role in dystrophic pathology (37) and the induction of trained immunity (72), therapeutic interference with this mechanism could be a promising avenue. In addition, given that the phenomenon of trained immunity is critically dependent on epigenetic rewiring of immune cells, interventions capable of modifying transcription factor interactions with the open chromatin state may also be considered (86). In this regard, it will be interesting to determine whether newly emerging or established drugs for DMD treatment such as givinostat (87) or corticosteroids (88), which are known to modulate epigenetic mechanisms, have an impact on the development of trained immunity in DMD. Finally, we speculate that the amplified innate immune response associated with trained immunity could alter the effectiveness of various gene therapy and other dystrophin restoration strategies for DMD.

## Author contributions

BP was the primary author of the manuscript and developed the major concepts proposed. TP helped to write the manuscript and develop the illustrative figures. SB helped to develop concepts in the manuscript. JT helped to develop the illustrative figures. JD helped to develop the figures and concepts outlined in the manuscript. All authors contributed to the article and approved the submitted version.
